# Industry Issues: Putting the Heat on Gas

**DOI:** 10.1289/ehp.115-a76

**Published:** 2007-02

**Authors:** Valerie J. Brown

The pace of energy development in the West is staggering. Since 1990 Colorado alone has seen a 450% increase in natural gas production, and now has more than 27,000 active gas wells, according to a fact sheet from the Western Colorado Congress, a community action group. This explosive growth puts the energy business into more frequent conflicts with traditional surface uses such as ranching, wilderness recreation, and housing developments. Thus, public exposure to the many chemicals involved in energy development is expected to increase over the next few years, with uncertain consequences.

Both air and water quality are affected by extraction of natural gas rich in methane. Sometimes methane must be separated from fluids and other gases in processes that emit volatile organic compounds (VOCs). Chemicals containing VOCs may also be used when a well is drilled and during a process known as hydraulic fracturing (“fracking”), in which chemical mixtures are injected into wells to break up rock formations and release gases. VOCs are also emitted by compressors and other equipment. “Produced water,” groundwater drawn from wells that can contain various salts as well as drilling and fracking chemicals, is usually reinjected underground or placed in evaporation ponds on the surface, from which chemicals including VOCs can be released to the atmosphere. Methane and fracking chemicals can also migrate into shallow aquifers used for drinking water wells.

Benzene, toluene, ethyl benzene, and xylenes are naturally present in many hydrocarbon deposits, and may be present in drilling and fracking chemicals. These VOCs can cause symptoms such as headache, loss of coordination, and damage to the liver and kidneys; benzene is a carcinogen as well. VOCs help create ground-level ozone, which can contribute to severe respiratory and immune system problems.

Although the EPA issued a 2004 report concluding there is very little risk that fracking can contaminate drinking water, there are some documented contamination incidents. For example, in August 2006, drilling fluids and methane were detected emerging from a hillside in Clark County, Wyoming, from a gas well surrounded by a rural housing development. Ultimately 8 million cubic feet of methane were released, according to the 17 November 2006 *Casper Star-Tribune*. Subsequent tests showed contamination of shallow groundwater with hydrocarbon compounds.

The drilling boom in Colorado’s Garfield County has triggered a rash of citizen complaints that petrochemical pollution has caused adrenal and pituitary tumors, headaches, nausea, joint pain, respiratory problems, and other symptoms. Half the state’s drilling rigs are in Garfield County, whose population is expected to increase by 62% over its 2000 census figure, according to the Colorado Department of Public Health and Environment. Because exposures to VOCs and other chemicals are largely unquantified, it is difficult to assess these claims. Still, VOC emissions in Garfield County rose 30% between 2004 and 2006, according to Mike Silverstein, deputy director of the Colorado Air Pollution Control Division. The county is currently conducting a health risk assessment and an ambient air quality monitoring study.

Theo Colborn, president of The Endocrine Disruption Exchange in Paonia, Colorado, believes that some drilling and fracking additives that can end up in produced water are neurotoxic; among these are 2-butoxyethanol. “If you compare [such chemicals] with the health problems the people have,” Colborn says, “they match up.”

Brian Macke, director of the Colorado Oil and Gas Conservation Commission (COGCC), says that group tested numerous wells after residents complained. “In any investigations we’ve made in Colorado, we’ve never determined there have been any impacts from any hydraulic fracturing operation by any of the constituent chemicals,” he says.

Activists counter that the COGCC has not tested specifically for the chemicals Colborn is concerned about, and that many drilling mud and fracking fluid recipes are proprietary and thus unavailable to the public. Without such tests, the true concentrations of these chemicals in produced water remain unknown, says Lisa Sumi, research director at the Oil and Gas Accountability Project in Durango.

Ken Wonstolen, senior vice president and general counsel of the Colorado Oil and Gas Association, denies that the industry threatens public health, but concedes that oil and gas emissions may be bothering some nearby residents. “There could be localized topography and certain atmospheric conditions so that some of the odors are pooling in a low area,” he says, adding that these “tend to be transient events.”

Most oil and gas industry emitters of VOCs are considered minor sources, and the EPA does not regulate them, says Silverstein. State and local agencies are now attempting to clarify the extent of industry emissions. In 2002 the Colorado Air Pollution Control Division conducted a preliminary survey of VOCs at several sites in Grand Junction, concluding that none of the chemicals measured posed “significant health risks to area residents.” Even so, reported the 18 December 2006 *Rocky Mountain News*, the Colorado Air Quality Control Commission voted to require VOC emission controls on more types of oil and gas equipment, and to require a 75% reduction of VOC emissions from certain gas storage tanks rather than the 47.5% reduction set in 2004 by the Pollution Control Division for the oil and gas fields near Denver.

## Figures and Tables

**Figure:**
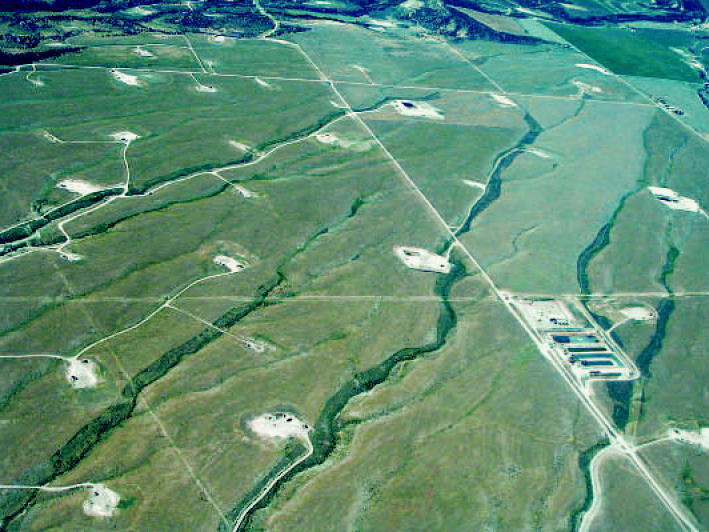
Petrochemical plats Natural gas wells dot the landscape in Garfield County, Colorado. Such sites are the subject of citizen concerns about their release of VOCs and other harmful chemicals into air and water.

